# Genetic parameters for mid-infrared spectroscopy–predicted fertility

**DOI:** 10.3168/jdsc.2021-0141

**Published:** 2021-09-23

**Authors:** I. van den Berg, P.N. Ho, M. Haile-Mariam, J.E. Pryce

**Affiliations:** 1 Agriculture Victoria Research, AgriBio, Centre for AgriBioscience, 5 Ring Road, Bundoora, Victoria, 3083, Australia; 2 School of Applied Systems Biology, La Trobe University, Bundoora, Victoria, 3083, Australia

## Abstract

Female fertility is a challenging trait to improve genetically because of its low heritability, its unfavorable genetic correlation with milk yield, and its relatively small number of records. The MFERT trait is the probability of conception to first insemination predicted using mid-infrared (MIR) spectroscopy of a milk sample collected during lactation as part of routine milk recording, age at calving, days in milk, and milk production. As such, MFERT could become available on many more cows than traditional fertility traits. Our objectives were (1) to estimate the heritability of MFERT; (2) to estimate genetic correlations between MFERT, traditional fertility traits, and milk production traits; and (3) to assess the potential of MFERT to be used as an indicator trait for fertility in a selection index. The MFERT trait had a heritability of 0.16, which was higher than that (0.05) obtained for traditional fertility traits. Genetic correlations between MFERT and traditional fertility traits were low to moderate. The weakest and strongest correlations (mean ± standard error) were with pregnancy at the end of the mating season (0.13 ± 0.05) and calving to first service (−0.61 ± 0.03), respectively. Based on our estimates, including MFERT in a fertility index will only substantially increase the accuracy of the index when there are many more records available for MFERT than for the traditional fertility traits. This is likely to be the case because the number of milk samples from commercial machines belonging to milk recording companies in Australia that are capable of generating MIR spectra is growing. Hence, the number of records for MFERT is expected to increase substantially in the near future.

## Highlights

• Mid-infrared (MIR)-predicted fertility has greater heritability than traditional fertility phenotypes.
• MIR-predicted fertility has moderate genetic correlations with traditional fertility phenotypes.
• Because MIR-predicted fertility mainly requires a milk sample, it could be available on many more cows than traditional fertility phenotypes.
• MIR-predicted fertility can increase the accuracy of a fertility index when available on a much larger number of cows than traditional fertility phenotypes.

## Graphical Abstract

**Figure:**
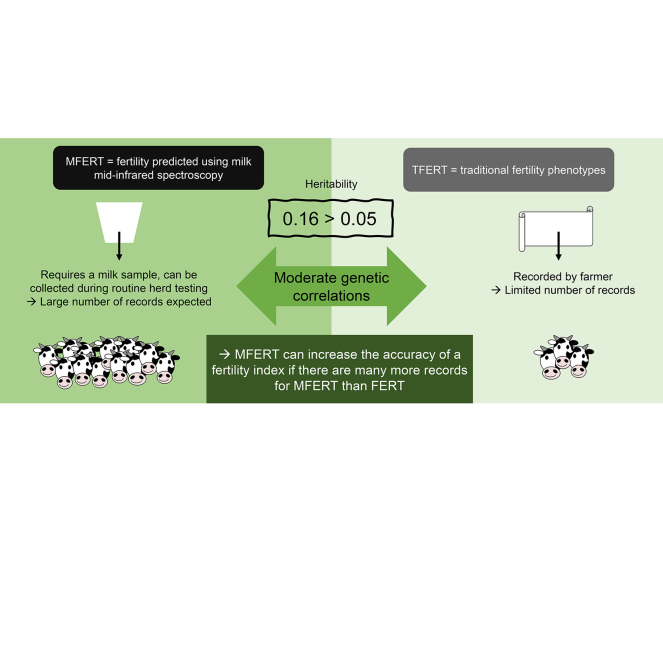
**Summary:** MFERT is the probability of conception to first insemination using mid-infrared spectroscopy of a milk sample collected during lactation as part of routine milk recording, age at calving, days in milk, and milk production. Because MFERT can be estimated during routine milk recording, it can become available on a much larger number of records than the traditional fertility phenotypes. To test the potential of MFERT for genetic selection, we estimated genetic parameters for MFERT. While MFERT had a higher heritability than traditional fertility phenotypes, genetic correlations between MFERT and traditional fertility phenotypes were low to moderate. The strongest genetic correlation was found with calving to first service, with a genetic correlation of -0.61. Therefore, MFERT will only be able to increase the accuracy of a fertility index if there are many more records for MFERT than for traditional fertility phenotypes.

Female fertility is an important trait in dairy cattle. Many studies have shown that it has been subject to declines due to its negative genetic correlation with production traits (Berry et al., 2014; Lucy, 2019). As a response to this, fertility has been a major part of the breeding objective worldwide over the last 20 yr (Miglior et al., 2005; Cole and VanRaden, 2018). Although this has led to favorable genetic trends in fertility (Berry et al., 2014), fertility remains the most common reason for culling in Australia (Workie et al., 2019). The low heritability of fertility (Ma et al., 2019) and unfavorable genetic relationships with other traits under selection limit the response to selection. Furthermore, variation in economic factors and genetic parameters between countries results in differences in the magnitude of response to selection (Pryce et al., 2014). Hence, it is more challenging for some countries to achieve positive genetic trends than for others.

The accuracy of EBV, whether they are predicted based on progeny records or genomic prediction, increases when the number of records for a trait increase. A larger number of progeny records increases the accuracy of the breeding value of a bull, and a larger number of records in the reference population used for genomic prediction increases the accuracy of genomic prediction (Goddard, 2009). This is especially true for traits with low heritability. If the reference population is sufficiently large, it can provide accurate breeding values for all genotyped bulls, including those with no or limited number of progeny records. Although fertility is routinely recorded for a large number of cows in Australia, the number of fertility records is smaller than that of other traits such as milk yield. Furthermore, unlike milk production data, most fertility data are not available when selection decisions are made. Having a fertility record for all cows with production records could help to substantially increase the reference population.

As part of routine milk recording, mid-infrared (**MIR**) spectroscopy is used to quantify the composition of milk, including fat, protein and lactose concentration. Recently, Ho et al. (2019) developed an equation to predict the probability of conception of dairy cows to first insemination (**MFERT**), using MIR data, age at calving, DIM, and milk production. MFERT was able to phenotypically classify cows into high or low fertile groups. The inference here is that by training the MFERT model that contrasts the most fertile cows (i.e., cows that conceived at first insemination) with the least fertile (i.e., cows with only one insemination within the mating season without conception), clearer biological signals might be observed and thus higher modeling accuracy could be obtained (Ho et al., 2019; Ho and Pryce, 2020).

Currently, the breeding objective of the fertility Australian Breeding Value (ABV) includes 6-week-in-calf rate derived from calving interval (**CI**), lactation length (**LL**), pregnancy at the end of the mating season (**PREG**), first-service nonreturn rate, and interval from calving to first service (**CFS**) using a 5-trait model (Haile-Mariam et al., 2013). We hypothesized that MFERT may be useful to be included as an additional trait for predicting the EBV for fertility. However, for this to be successful for genetic improvement of fertility, MFERT should be heritable, significantly genetically correlated with other fertility traits already in the breeding objective, and be available in a large quantity. Currently, the genetic parameters of MFERT are unknown.

The negative genetic correlation between milk yield (**MY**) and fertility also means that MY could be used as an indicator trait to predict fertility (Biffani et al., 2005; Harris et al., 2005). Milk yield has a higher heritability than fertility and is available on a larger number of cows than fertility traits. However, including MY for predicting fertility may unintentionally mean selecting for lower MY; as such (although of interest to include in this study), it is unlikely to be considered as a selection criterion for national evaluations of fertility. As MFERT is derived from a milk sample, understanding genetic correlations with milk production traits is also important. Our objectives were (1) to estimate the heritability of MFERT; (2) to estimate genetic correlations among MFERT, traditional fertility traits, and MY; and (3) to compare the potential of MFERT to be used as indicator trait for fertility in a selection index with MY.

Table 1 summarizes the data used for our analyses. Phenotypes for traditional fertility traits (**TFERT**) including CI, LL, CFS, and PREG, as well as test-day MY, test-day fat yield (**FY**), test-day protein yield (**PY**), test-day fat percentage (**F%**), and test-day protein percentage (**P%**) were provided by DataGene (Bundoora, Victoria, Australia), for 618,856, 598,732, 357,110, 302,069, 642,617, 641,340, 641,339, 641,341 and 641,399 Holstein cows with records for CI, LL, CFS, PREG, MY, FY, PY, F%, and P%, respectively, with one record per cow. Depending on the trait, between 3,157 (MFERT) and 39,177 (MY) cows with phenotypes had imputed genotypes for the Illumina Bovine HD BeadChip (**HD**). More details on the genotyping and imputing pipeline are described by van den Berg et al. (2021).

**Table 1:** Number of cows with records (nP) and genotypes (nG) and the mean and standard deviation (SD) of each trait

Acronym	Trait	nP	nG	Mean	SD
CI	Calving interval (d)	618,856	35,014	410	61
LL	Lactation length (d)	598,732	34,050	360	59
CFS	Calving to first service (d)	357,110	34,899	93.7	46.30
PREG	Pregnancy (0,1)	302,069	30,633	0.9	0.36
MFERT	Probability of conception to first insemination predicted using MIR spectroscopy (0, 1)	4,124	3,157	0.8	0.12
MY	Test-day milk yield (L)	642,617	39,177	27.6	8.07
FY	Test-day fat yield (kg)	641,340	39,107	1.0	0.34
PY	Test-day protein yield (kg)	641,399	39,111	0.9	0.25
F%	Test-day fat percentage (%)	641,340	39,107	3.7	0.75
P%	Test-day protein percentage (%)	641,399	39,111	3.1	0.32

The MFERT trait is the probability of conception to first insemination predicted using milk MIR, age at calving, DIM, and MY, derived using the model described in detail by Ho et al. (2019) and Ho and Pryce (2020). Data used for our study were collected between 2016 and 2018 (inclusive) from 29 commercial Australian dairy herds. The MIR spectra were obtained from the analysis of milk samples at Hico Pty Ltd. (Maffra, Victoria, Australia), TasHerd Pty Ltd. (Hadspen, Tasmania, Australia), or DairyExpress (Armidale, New South Wales, Australia) using NexGen Series FTS Combi machines (Bentley Instruments). Other data such as age at calving, DIM at herd testing, and milk production were obtained from DataGene. The MIR spectra used in our analyses were recorded between 0 and 265 DIM, with 97% of MFERT records being within 150 DIM.

To independently obtain the value of MFERT for cows in each herd, we applied a procedure that is similar to external validation. To achieve this, we predicted the probability of conception of cows (ranging from 0 to 1) in each herd in turn by applying a model that was trained using data from the other 28 herds. Hence, the model was completely independent from the herd being predicted. In this study, we only used predicted probabilities of Holstein cows. Where a cow had multiple records within a lactation, only the first record was selected. This resulted in 4,124 cows with MFERT records.

Genetic parameters were estimated using GIBBS2F90 (Misztal et al., 2014). A combined pedigree (constructed using 3 generations of pedigree) and genomic relationship matrix (**H**) was constructed following Aguilar et al. (2010), using genotypes of 576,431 variants on the HD chip. A univariate animal model fitting fixed effects (herd-year-season, age within parity, and month of calving), alongside **H** and a vector of random residuals, was used to estimate heritabilities. A bivariate model with the same effects was used to estimate genetic correlations between traits.

To assess the potential advantage of including MFERT in a fertility index, we compared the accuracy of the EBV of a bull for several indices. The basic index contained CI, LL, CFS, and PREG and is referred to as FERT*n_f_*, where *n_f_* is the number of progeny records for CI, LL, CFS, and PREG, with *n_f_* = 0, 10, 30, or 60 for each of the TFERT traits. The accuracy of the FERT index was compared with indices with *n_f_* records for FERT and 60 for MFERT (FERT*n_f_*+MFERT60), MY (FERT*n_f_*+MY60), or MFERT and MY (FERT*n_f_*+MFERT60+MY60). The accuracy of each index was calculated using software developed by J. van der Werf (https://jvanderw.une.edu.au/software.htm ), following Mrode (2005):

$$r_{IH}=\frac{\sigma_{I}}{\sigma_{H}},$$

where $\sigma_{I}$ is the standard deviation of the index and $\sigma_{H}$ the standard deviation of the breeding objective. In our indices, the breeding objective was always CI. All other traits were used as selection criteria; that is, as correlated traits with economic weights of 0. Hence, the standard deviation of the breeding objective equaled the standard deviation of CI.

The heritability of MFERT was substantially larger than that of the fertility traits currently included in the Australian selection indices (0.16 vs. 0.05, respectively; Table 2). Heritabilities for TFERT were in line with the report of Ma et al. (2019). As a consequence of the number of records available, the standard error of the heritability of MFERT was larger than that of TFERT. Standard errors were 0.03 for MFERT and ≤0.01 for TFERT. Despite the relatively large standard error, the heritability of MFERT was more significant than that of TFERT. This means that genetic improvement of MFERT may be easier to achieve than that of TFERT. MFERT might have a higher heritability than the other fertility traits because some of the factors that contribute to the residual variance of TFERT. For example, culling and recording errors may affect MFERT less than TFERT. Furthermore, the trait MFERT predicts the probability of conception and may be a more precise and therefore more heritable phenotype than TFERT.

**Table 2:** Heritabilities (h^2^), genetic correlations with MFERT (r_g,MFERT_), MY (r_g,MY_), F% (r_g,F%_), and P% (r_g,P%_), and standard errors (SE)^1^

Trait	h^2^ ± SE	r_g,MFERT_ ± SE	r_g,MY_ ± SE	r_g,F%_ ± SE	r_g,P%_ ± SE
CI	0.05 ± 0.01	−0.32 ± 0.04	0.44 ± 0.02	−0.03 ± 0.03	−0.23 ± 0.02
LL	0.05 ± 0.00	−0.36 ± 0.03	0.45 ± 0.03	−0.08 ± 0.02	−0.28 ± 0.02
CFS	0.05 ± 0.01	−0.61 ± 0.03	0.43 ± 0.03	−0.06 ± 0.02	−0.24 ± 0.02
PREG	0.05 ± 0.01	0.13 ± 0.05	−0.08 ± 0.04	−0.04 ± 0.05	0.06 ± 0.04
MFERT	0.16 ± 0.03	—	−0.32 ± 0.05	0.50 ± 0.05	0.46 ± 0.04
MY	0.11 ± 0.01	−0.32 ± 0.05	—	−0.52 ± 0.01	−0.53 ± 0.01
FY	0.08 ± 0.00	0.24 ± 0.06	0.37 ± 0.02	0.59 ± 0.01	0.07 ± 0.02
PY	0.07 ± 0.00	−0.14 ± 0.04	0.82 ± 0.00	−0.26 ± 0.02	0.04 ± 0.02
F%	0.14 ± 0.01	0.50 ± 0.05	−0.52 ± 0.01	—	0.53 ± 0.01
P%	0.16 ± 0.00	0.46 ± 0.04	−0.53 ± 0.01	0.53 ± 0.01	—

1 CI = calving interval; LL = lactation length; CFS = calving to first service; PREG = pregnancy; MFERT = mid-infrared spectroscopy predicted fertility; MY = test-day milk yield; FY = test-day fat yield; PY = test-day protein yield; F% = test-day fat percentage; and P% = test-day protein percentage.

MFERT had significant genetic correlations with all other traits. The weakest correlation was found with PREG (0.13) and the strongest correlation with CFS (−0.61; Table 2). Based on the trait definitions, we expected MFERT to be closer to PREG than CI, LL, and CFS: MFERT predicts the probability of conception, whereas PREG measures the outcome of conception. However, genetic correlations were stronger with CI, LL, and CFS than with PREG. This may be because, like MFERT, CI, LL, and CFS are all continuous traits, whereas PREG is a binary trait. Because the strongest correlation was detected between MFERT and TFERT, adapting MFERT to predict CFS rather than PREG may further increase the genetic correlation between MFERT and CFS. Furthermore, MFERT is better at predicting high and low fertile cows than moderately fertile cows (Ho et al., 2019; Ho and Pryce, 2020). If we assume a normal distribution for fertility, moderately fertile cows would make up a substantial proportion of our data. This may have contributed to the relatively low genetic correlations between MFERT and TFERT.

Similar to TFERT, MFERT had a moderate, unfavorable genetic correlation with MY. However, the correlation between MFERT and MY (−0.32) was lower than that between MY and CI, LL, and CFS (0.43 to 0.45). Consequently, the correlated response to selection for MY was −2.1 kg with the FERT0+MY60 index and −0.68 kg with FERT0+MFERT60. Hence, using MFERT as predictor fertility trait has a smaller effect on MY than using MY as predictor. Genetic correlations between MFERT were larger with F% (0.50) and P% (0.46) than with FY (0.24) and PY (−0.14). There has been considerable interest in protein percentage as a predictor of fertility genetically (Morton et al., 2018). Douglas et al. (2016) showed that cows with high milk protein concentration have higher plasma profiles of several metabolites and hormones, which could indicate energy partitioning pathways. Both F% and P% are, like MFERT, derived from MIR spectra. The genetic correlations between MFERT and TFERT were stronger than those between F% or P% and TFERT. Indeed, Ho et al. (2019) reported that adding MIR spectra significantly increased model accuracy compared with using only milk composition. The underlying biology of MFERT is not, however, well understood currently.

Figure 1 compares the accuracies of different fertility indices. Using only TFERT (CI, LL, CFS, PREG) yielded accuracies of 0.42, 0.62, and 0.75 using 10, 30, or 60 progeny records for each trait, respectively. In the absence of any TFERT records, having 60 progeny records for MFERT, MY, or both yielded accuracies of 0.26, 0.35, and 0.40, respectively. Including MFERT, MY, or both in the index alongside TFERT increased the accuracy. The largest increases in accuracy were observed when only 10 fertility progeny records were used. The FERT10+MFERT60 index yielded an accuracy 0.45; FERT10+MY60 had a higher accuracy, of 0.52; and FERT10+MFERT60+MY60 further increased the accuracy to 0.53. With 30 fertility progeny records, increases were smaller. When 60 fertility records were available, FERT60 and FERT60+MFERT60 resulted in the same accuracy, and only a small increase was observed for FERT60+MY60 and FERT60+MFERT60+MY60. In all scenarios, MY was more beneficial than MFERT as an indicator trait for CI. This was expected, because the genetic correlation between MY and CI was stronger than the correlation between MY and MFERT (Table 2).

**Figure 1:**
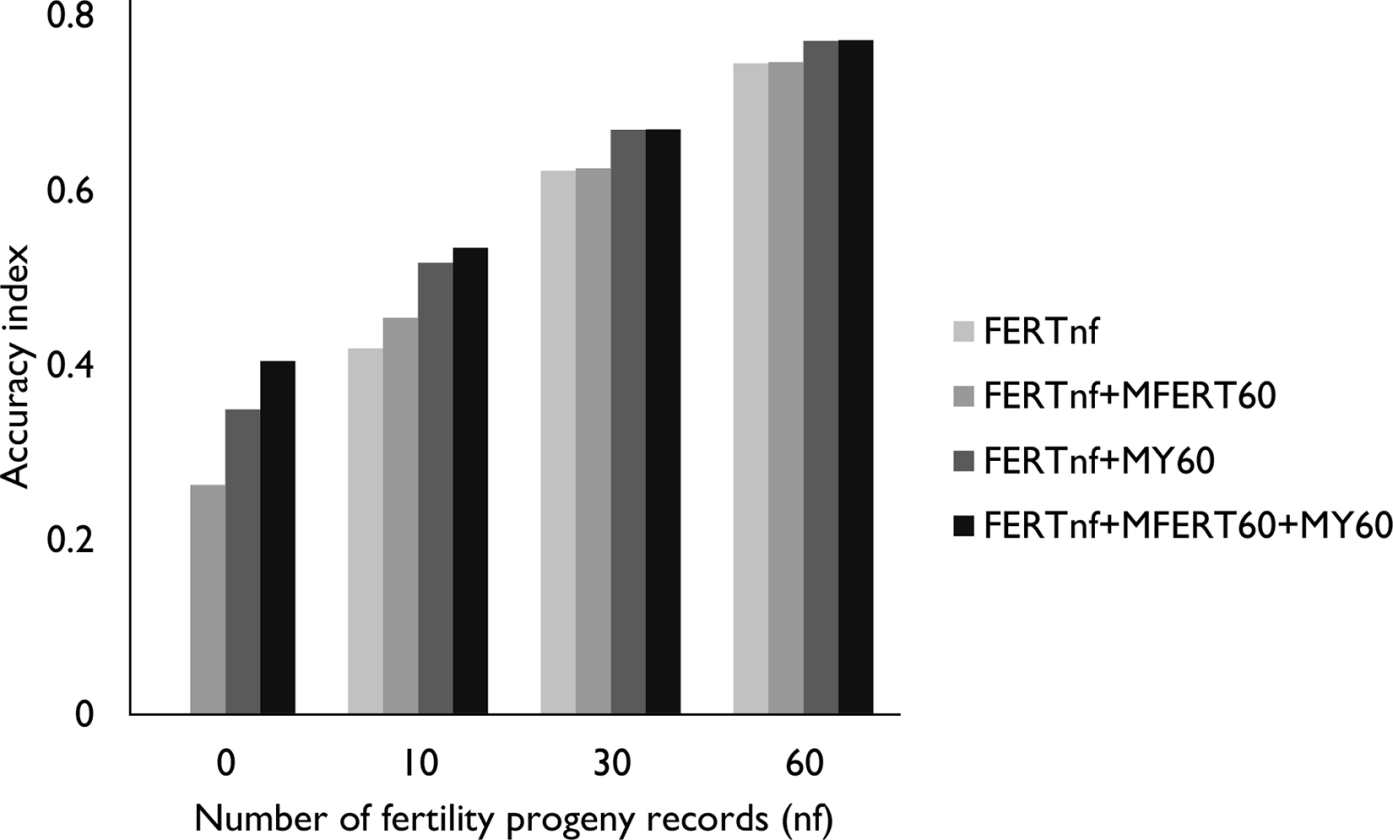
Accuracies of selection indices. Indices contained fertility traits calving interval, lactation length, calving to first service, and pregnancy (FERTnf), fertility traits and mid infrared (MIR)-predicted fertility (FERTnf+MFERT60), fertility traits and milk yield (FERTnf+MY60), or fertility traits, MIR-predicted fertility, and milk yield (FERTnf+MFERT60+MY60). The number of progeny records available for FERT (nf) was 0, 10, 30, or 60, and that for MFERT and MY was 60.

The main appeal of MFERT for use as an indicator trait for fertility is that it requires only a milk sample. Hence, it could be available for all cows that go through routine herd testing. Additionally, MFERT could be available as soon as a cow starts lactating, which is earlier than most TFERT. While having MFERT records obtained routinely through herd testing would result in a much larger number of records for MFERT than for TFERT, the current situation in Australia is the reverse: we have many more records for TFERT than for MFERT. Therefore, the benefit of using MFERT for genetic selection is limited at the moment. However, MFERT is a promising tool for phenotypic prediction and management (Ho et al., 2019; Ho and Pryce, 2020), and MIR samples are being collected and used for prediction of a wide range of other traits (e.g., Ho et al., 2021). Consequently, the number of records for MFERT is expected to increase substantially in the near future as MIR data become routinely collected and stored from milk recording organizations across Australia. When more data for MFERT become available, we recommend re-estimating the genetic parameters on a larger data set and subsequently reassessing the potential benefit of including MFERT in a fertility index. In addition to more animals with records, having more records per cow, both within lactation and across lactations, may also help improve the accuracy of the MFERT prediction equations, the estimation of genetic parameters, and genomic prediction.
